# Editorial: Pathogenesis of Dimorphic Fungal Infections

**DOI:** 10.3389/fcimb.2021.793245

**Published:** 2021-11-11

**Authors:** Angel Gonzalez, Carlos P. Taborda

**Affiliations:** ^1^ School of Microbiology, Universidad de Antioquia, Medellín, Colombia; ^2^ Institute of Biomedical Sciences, Department of Microbiology and Institute of Tropical Medicine, Laboratory of Medical Mycology (LIM53), University of São Paulo, São Paulo, Brazil

**Keywords:** fungal pathogenesis, dimorphic fungi, Histoplasma, Paracoccidioides, Coccidioides, Blastomyces, Talaromyces, Emergomyces

## Pathogenesis of Dimorphic Fungal Infections

Dimorphic fungal infections are responsible for the development of life-threatening diseases, especially in patients with a compromised immune system that include those infected with the human immunodeficiency virus (HIV) or those receiving antineoplastic agents, immunosuppressive agents used in solid organ receptors, immunomodulatory therapies, and other biological products. Also, the progressive devastation of tropical forests changing the entire balance of nature is responsible for increase of endemic mycoses. These infections are caused by dimorphic fungi belonging to species of the genus *Histoplasma*, *Paracoccidioides*, *Coccidioides*, *Blastomyces*, *Talaromyces*, and *Emergomyces*, which are distributed in defined geographical areas. The mortality and morbidity caused by these mycoses have increased rapidly during the last decades, especially in countries where infections, by these fungi, are endemic. In this Research Topic you will find a number of reports aiming at a better understanding of the pathogenesis of dimorphic fungal infections and all the factors involved in both the host and the causative agent. Contributions focus mainly on virulence factors of fungal agents, host-pathogen interactions, risk factors in the host, immune response, and diagnosis.

The study conducted by Assunção et al. describes some aspects of the nutritional immunity and how the response is of *Histoplasma capsulatum* under zinc (Zn) deprivation through genomic and proteomic analysis. These authors report that this fungus harbor eight genes related to Zn homeostasis, and the expression of ZAP1, ZRT1, and ZRT2 is induced under zinc-limiting conditions; moreover, during zinc deprivation, proteins related to energy production pathways, oxidative stress, and cell wall remodeling were regulated, as well, increase in chitin and glycan content in fungal cell wall was observed.

Among the different virulence factors in *H. capsulatum*, Fregonezi et al. describe that blocking a heat shock protein of 60 kilodaltons (Hsp60) with a monoclonal antibody reduces the metabolic activity and biomass of this pathogen, and in addition, this blockage increases the survival of the larvae *Galleria mellonella* after infection, thus indicating that this Hsp60 participates in both the biofilm formation and pathogenesis. Furthermore, Gonçalves et al. describe that *H. capsulatum* produce cellular-attached (C-gly-*Hc*) and secreted (E-gly) glycans with reactivity to glucuronoxylomannan (GXM) monoclonal antibodies developed against GXM from *Cryptococcus neoformans*; noteworthy, this GXM-like *Hc* glycans also react with sera of cryptococcosis patients, and additionally, acapsular *C. neoformans* (cap59 strain) covered with this GXM-like *Hc* glycans were more resistant to phagocytosis and macrophage killing and increase death rates of *G. mellonella* larvae, suggesting that these molecules are important during host-fungal interactions.

In *Paracoccidioides brasiliensis*, a study addressed by Zonta et al. reported that this pathogen releases a DNase-like protein that degrades neutrophil extracellular traps (NETs) that allows its fungal escape. On these lines, Longo et al. also demonstrated that *P. brasiliensis* produces a unique 1-Cys peroxiredoxin, which is localized both in the cytoplasm and cell wall, which confer it the capability to decompose hydrogen peroxide, one of the most abundant and antioxidant compounds produced by the host in response to infection. Furthermore, Almeida Donanzam et al. inform that exoantigens from *P*. *brasiliensis* and *P. lutzii* strains induce significant proliferation of both human and murine pulmonary fibroblast and increased levels of the pro-fibrotic cytokine TGF-β1 and pro-collagen type I, suggesting that these fungal components participate in the fibrogenesis process induced by these fungal pathogens.

In *Coccidioides*, Peláez-Jaramillo et al. conducted an elegant lipidomic work; in this study, the authors demonstrated that this fungal pathogen secrets a lipid-rich, membranous cell surface layer, *in vivo* and *in vitro*, composed mainly by phospholipids [acylglycerols and sphingolipids (sphingosine and ceramide)] and saturated fatty acids (myristic, palmitic, elaidic, oleic, and stearic acid), which contribute to the suppression of inflammatory response and the subsequent dissemination of the fungal infection.

Danchik and Casadevall describe that cell surface hydrophobicity (CSH) is an important cellular and biophysical parameter that affects both cell-cell and cell-surface interactions; and in dimorphic fungal pathogens, this parameter can be affected by multiple variables including altered cell wall composition, genetic modification, changes in temperature, and altered nutrient availability that in turn could affect, indirectly, several biological process such as biofilm formation, virulence, and response to antifungal treatments.

Other important aspect that allows dimorphic fungal pathogens to survive inside macrophages is their intracellular metabolism; thus, the fungal growth, proliferation, and survival within macrophages require strategies for acquisition of sufficient nutrients from the nutrient-depleted phagosomal environment. On these lines, Shen and Rappleye, after a rigorous review of transcriptomic and functional genetic studies in *Histoplasma* and *Paracoccidioides*, describe how these fungal dimorphic pathogens activate their metabolism to the resources available in the macrophage phagosome. Noteworthy, Giusiano G. describes an interesting mechanism called the “Trojan horse model” in which *Paracoccidioides* persists, as a facultative intracellular pathogen, within phagocytes, and allows it to transmigrate and disseminate to other tissues; thus, this mechanism, also reported for other fungi, is considered a factor of pathogenicity.

Regarding the immune response developed by the host against these fungal pathogens, Diep and Hoyer compilate several studies and resume the innate and adaptive immune response against *Coccidioides* infection. Of note, Jannuzzi et al. contribute with an interesting review highlighting the role played by intracellular pattern recognition receptors [(PRRs)—TLR3, TLR7, TLR8, and TLR9—which recognize mainly genetic material] both in controlling the infection and in the host’s susceptibility against important fungal infections, including paracoccidioidomycosis. Interestingly, de Oliveira et al., using an experimental model of PCM, observed that β2 integrin appears to play an important role in fungal survival inside macrophages, which serve as a protective environment for this fungal pathogen. Additionally, Puerta-Arias et al., after an exhaustive review of several studies, also describe that T-cell-mediated immunity, mainly T helper (Th)1 and Th17 responses, is essential for protection against the majority of the dimorphic fungi; thus, IL-17 production is associated with neutrophil and macrophage recruitment at the site of infection accompanied by chemokine and pro-inflammatory cytokines, a mechanism mediated by some PRRs and adaptor molecules, which in turn play distinctly different roles for each pathogen, being beneficial mediating fungal controls or detrimental promoting disease pathologies.

Kischkel et al. contribute with an important review concerning the use of nanoparticles to develop new therapeutic options, including vaccination against systemic mycosis including those caused by *Candida* spp., *Cryptococcus* spp., *Histoplasma* spp., *Paracoccidioides* spp., *Coccidioides* spp., and *Aspergillus* spp.; these authors provide important information about the use of different types of nanoparticles, nanocarriers, and their corresponding mechanism of action.

With respect to the diagnosis of dimorphic fungal infections, Almeida et al. inform the development of a co-immunoprecipitation assay using a protein extract from the yeast morphotype of *H. capsulatum* and pooled sera from patients with proven histoplasmosis, followed by a shotgun mass spectrometry identification of antigenic targets; the authors found three antigens as potential antigenic targets (M antigen, catalase P, and YPS-3), and 16 regions from these three proteins were proposed as putative B-cell epitopes exclusive to *H. capsulatum*; they indicate their possible use in new methods for the diagnosis of histoplasmosis.

Noteworthy, Nacher et al. contribute with two reports; in the first one, they describe three cases of HIV patients with disseminated histoplasmosis who had adrenal insufficiency, a presentation that is not common in these types of immunosuppressed patients. In the second contribution, Nacher et al. described a series of cases of patients with advanced HIV and disseminated histoplasmosis with superficial and deep lymphadenopathies; of interest, the presence of deep lymphadenopathies was associated with fewer biomarkers of severity and a lower risk of death.

Finally, Drak Alsibai et al. contribute with a report of 15 years of experience of a Pathology Center of French Guiana; the authors made a cytological and histopathological analysis of samples from patients with disseminated histoplasmosis, the largest series to date, and described that digestive involvement was the most frequent, usually with tuberculoid form a greater load of fungal cells, and concluded that cytology and pathology are widely available methods that can give life-saving results in a short time to help orient clinicians facing a potentially fatal infection requiring prompt treatment.

The above contributions to this Research Topic highlight recent advances and increase our knowledge about the complex host-fungal interactions and how through several virulence attributes, dimorphic fungal pathogens are able to survive in different environments, especially into the mammalian host, and how they overcome the immune response. Understanding these mechanisms could provide novel targets for implementing new therapeutic strategies or intervention.

## Author Contributions

AG and CT edited the topic and wrote the manuscript. All authors contributed to the article and approved the submitted version.

## Conflict of Interest

The authors declare that the research was conducted in the absence of any commercial or financial relationships that could be construed as a potential conflict of interest.

## Publisher’s Note

All claims expressed in this article are solely those of the authors and do not necessarily represent those of their affiliated organizations, or those of the publisher, the editors and the reviewers. Any product that may be evaluated in this article, or claim that may be made by its manufacturer, is not guaranteed or endorsed by the publisher.

